# London Education and Inclusion Project (LEIP): Results from a Cluster-Randomized Controlled Trial of an Intervention to Reduce School Exclusion and Antisocial Behavior

**DOI:** 10.1007/s10964-016-0468-4

**Published:** 2016-03-23

**Authors:** Ingrid Obsuth, Alex Sutherland, Aiden Cope, Liv Pilbeam, Aja Louise Murray, Manuel Eisner

**Affiliations:** Institute of Criminology, University of Cambridge, Sidgwick Site, Cambridge, CB3 9DA UK

**Keywords:** School exclusion, Cluster-randomized controlled trial, School-based intervention, Adolescence

## Abstract

School exclusion as a disciplinary measure remains a controversial issue. In spite of numerous attempts to reduce this practice, no solutions with documented effectiveness exist. This article reports results of a cluster-randomized controlled field trial carried out in 36 schools across London. The trial is an independent evaluation of a 12-week-long intervention, Engage in Education-London (EiE-L), delivered by Catch22. The intervention was aimed at students in secondary school who are most at risk of school exclusion. It targeted their social communication and broader social skills with the aim of reducing school exclusions and problem behaviors. The study employed a multi-informant design that included students and teacher reports as well as official records for exclusions and arrests. Data were analyzed through intent-to-treat analyses based on self-reports from 644 students and 685 teacher reports for students who were nominated for the study and for whom data was available at baseline or post-intervention. At baseline data collection the students ranged in age from 12.85 to 15.03, with *M* = 14.03; 71 % were male and included a number of ethnic minorities, the largest of which was black African/black Caribbean comprising 40 % of the sample. The results suggested a small but statistically significant negative effect on the primary outcome of exclusion and null effects for the secondary outcomes that measured behavioral and socio-emotional outcomes. The study’s findings are discussed in terms of the possible reasons for the null effects and negative (iatrogenic) effect.

## Introduction

Fixed period and permanent exclusions are used in schools in the United Kingdom as a method of tackling the more severe forms of student misbehavior, such as physical violence, or persistent disruptive behavior. Exclusion (also known as suspension in other jurisdictions such as the United States) is used to remove disruptive students from classrooms on a temporary or permanent basis. However, research suggests that exclusions are associated with poor academic and occupational outcomes, externalizing behavior (such as criminal activity), and internalizing behavior problems (such as self-harm; e.g., Gazeley et al. 2013; Lanskey 2015). High proportions of juvenile offenders and prisoners report having been excluded from school prior to being convicted, suggesting that exclusion is situated somewhere on a trajectory to later offending and incarceration for many students (e.g., Challen and Walton 2004). Furthermore, a recent study by Perry and Morris (2014) found that students attending schools that exclude more frequently than other schools appear to suffer academically, *whether or not those students are excluded*. This is in contradiction to an often cited justification for exclusion as a policy, namely that the disruption caused to other children is unfair and risks their educational achievement (e.g., Noguera 2003; Perry and Morris 2014).

Punishment for misbehavior at school is the first time that many children are sanctioned outside of the home. How this is carried out by the school and perceived by the child may have important consequences for later life, but more immediately for their socio-emotional development and educational attainment. Students who are excluded may show escalations in the negative behaviors that led to the exclusion if they perceive this sanction as unfair (e.g., Piquero et al. 2004), feel stigmatized by being excluded and/or feel no, or deny feeling, shame about being excluded. In addition, by being labelled as a “bad guy”, students may identify themselves with this label and through a self-fulfilling prophecy (Bernburg and Krohn 2003) engage (more) in the behaviors that originally led to this label. Further, by being excluded from school, adolescents may also have more opportunities to spend time in environments conducive to crime (e.g., Wikström et al. 2012). Exclusion from school is also the most explicit form of rejection by the educational system (Munn and Lloyd 2005). Therefore, there is also a risk that exclusion could weaken students’ perhaps already fragile relationships and engagement (bond) with school, through removing the fear of punishment and/or making them feel rejected. Either way, exclusion signals that further help may be needed by the student and/or the school.

What also calls into question the defensibility of relying on exclusion as a sanction for misbehavior is that, in the case of fixed-period exclusions, students in England and Wales have few demands placed on them while excluded, and receive minimal support upon returning to school. Schools are required to set and mark work for exclusions lasting more than one day but are only required to arrange alternative education after the fifth day of a fixed-period exclusion. While guidelines require schools to have a strategy for the reintegration of students upon return to school after a fixed period exclusion, there is no further clarification on what this should constitute. Moreover, there are no mechanisms in place to check the degree to which these guidelines are followed (Department for education; DfE 2012). For policy-researchers, this means that the deep irony of exclusion as a “punishment” is that for some children who are not bonded to school, exclusion is viewed as nothing more than a school sanctioned “holiday” (Dupper et al. 2009).

Children and adolescents at the highest risk of school exclusion experience a variety of vulnerabilities, including mental health problems, learning difficulties, experiences of maltreatment in and outside of the home, poverty, and other risk factors. Students who are excluded tend to be “hard to reach”, disruptive and in many cases aggressive toward adults and/or other peers. Exclusions are also not meted out to all students equally. Over-represented groups include male students, students from low socio-economic groups, students with special educational needs, and ethnic minorities (e.g., Gazeley et al. 2013; Office of the Children’s Commissioner (OCC) 2012; OCC 2013). Those excluded may not like school in the first place, perhaps partly as a result of finding school difficult due to their (unmet) educational needs (DfE 2012). Moreover, while official records are kept for permanent exclusions, fixed-period exclusions in the UK have been less systematically monitored or entirely unrecorded at times (Osler and Hill 1999), leading to underestimates in the numbers of exclusions. Furthermore, the issue of “illegal” and unrecorded exclusions complicate attempts to understand the full impact of exclusions (OCC 2013).

In summary, exclusion is widely used in the UK, but evidence suggests that it is an ineffective—and even potentially harmful—way of dealing with students with problematic behavior (Gazeley 2010; Osler and Vincent 2003). While interventions targeting behavior problems and school exclusion in youth exist and are implemented in many schools, few of them have been subjected to a rigorous evaluation. It is therefore not clear if and to what extent they are effective. For this reason, in the current study we evaluated a pre-existing intervention that aimed to decrease school exclusions and related problem behaviors.

### The Intervention

To procure an intervention for this evaluation, the research team approached the Education Endowment Foundation (EEF), which specialises in funding randomized controlled trials (RCTs) in school settings. A bidding process organised by the EEF sought to identify a suitable program and provider and drew up a shortlist of potential interventions. Catch22 and its Engage in Education—London (EiE-L) program was selected for evaluation as it had the clearest description of aims and mechanisms of change, and also presented promising findings from a preliminary evaluation (see Catch22 2013). In addition, Catch22 has delivered a range of programs to high-risk youth of varying ages in the UK.

The EiE-L intervention aimed to improve students’ behavior by developing their communication and broader social skills. EiE-L operated at the individual, school and family level. It aimed to provide targeted support to students with issues they were particularly struggling with, support teachers in addressing the behavioral and communication needs of students, and assist families to better support their children in school. The program consisted of one-hour long group and one-to-one sessions with students over 12 weeks. Each group session was delivered by two core-workers who were assigned to a school and one-to-one sessions were delivered by one of these core-workers. Based on prior experience working with youths, twelve core-workers were recruited and employed on a one year contract specifically to deliver this intervention. Core workers attended a four-week long training program on the principles and delivery of EiE-L. Material for group sessions was developed in conjunction with I CAN, a specialist communication charity, and addressed different aspects of communication difficulties (understanding, language processing, expressive language, and social communication) as well as social and behavioral issues. Session content and the resources required for delivering each session (e.g., scheme of work, session plans, session worksheets) were described in a guidebook available to each core worker at the time of the training. Sessions focused on interpersonal social skills such as effective anger management skills, assertive communication skills, or learning to appreciate the availability of different response alternatives in a variety of situations. See Table 1 for the description of the curriculum and main goals of each of the 12-sessions. One-to-one sessions were used to build on themes covered in group sessions or help participants with specific problems at home or at school. Home visits or phone calls to parents allowed the intervention providers to maintain engagement in the intervention by informing parents or carers about the performance of their child, positive or negative. Finally, I CAN provided support for teaching staff by delivering training sessions, conducting observations and conducting additional follow-up sessions. Please see the study protocol (Obsuth et al. 2014) for a full description of the intervention.

**Table 1:** EiE-L session goals and description

Sessions	Main contents
1. The skills I start with	To learn effective communication skills. Participants are invited to think about their strengths and difficulties in regard to their communication strategies with teachers and peers
2. Managing difficult emotions	To learn effective anger management skills. Participants are made aware of a range of emotions, the triggers for some emotions and some alternatives for managing them
3. Understanding conflicts	To learn strategies for self-calming and de-escalating confrontations
4. I have choices	To learn to appreciate the availability of different alternatives in a range of situations, to appreciate choices; their causes and effects
5. Check it out	To learn to identify difficulties in comprehension; being aware of confusion by instructions; positive skills and attitudes to ask for extra explanations (e.g., interrupting appropriately)
6. Different talk for different people	To learn to adjust the way of talking depending on one’s conversation partner and location. Develop an understanding of the difference between formal and informal communication exchanges
7. Looking back looking forward	Evaluate personal performance and setting goals for the second part of the course
8. Co-operating with others	To learn assertive communication skills in-group situations. Discussing with others in small groups, accepting others’ opinion, changing personal opinions
9. Aggressive, assertive, passive	To learn to understand and be aware of different styles of communication (aggressive, assertive, passive) and develop skills for adaptive, assertive interchange
10. Communication without talk	To learn to understand body language and non-verbal signals. To be aware of potential biases based on non-verbal signs/stereotypes (dress, ethnicity, posture, etc.)
11. I can change my world	To learn to identify and acknowledge personal difficulties with classroom behavior and identify strategies to improve
12. Summing up	Final session summarizing the learning process, relevance of communication skills, personal achievements and personal challenges

Table reproduced from published study protocol (Obsuth et al. 2014)

### Theory and Research Supporting the Intervention

The focus on the social aspects of communication and broader social skills represented the theory of change endorsed by the intervention provider. This theory of change appeared plausible in the context of other research suggesting that students who are excluded often have social-skills and social communication difficulties which may compromise their ability to benefit from the curriculum and behave prosocially (Clegg et al. 2009). Links between social, cognitive and interpersonal communication difficulties and behavioral problems at school have been identified in the literature. Researchers suggest that social-cognitive processes such as social communication problems (e.g., Gilmour et al. 2004; Moffitt and Scott 2009), social-emotional learning difficulties (Durlak et al. 2011), agency skills (e.g., Larsen and Angus 2011) and deficient social competence (Dodge et al. 1986), and/or hostile-attribution biases and problem solving (Dodge et al. 2006, 2013) facilitate the development and maintenance of antisocial-behavior problems. A broader understanding that social-cognitive and emotional skill development from childhood through adolescence are important for long-term success (e.g., Organisation for Economic Co-operation and Development; OECD 2015).

Several meta-analyses have demonstrated the positive effects of social-skills based programs on reducing aggressive and disruptive behavior (Sandler et al. 2014). For example, two meta-analyses examined the effectiveness of similar interventions to EiE-L which focused on social skills (Beelmann and Lösel 2006). Both studies identified small, but significant effects on anti-social behavior at the post-intervention assessment as well as long-term follow-up (Beelmann and Lösel 2006). Beelman and Lösel (2006) also examined the effects of these programs in different age groups and found that the effects were largest in adolescence (from age 13 and up; d = .61). In another meta-analysis, Derzon et al. (2006) detected small but significant effects of social-skills based programs on reducing physical violence and criminal behavior. Similarly, in a meta-analysis by Mytton et al. (2006), the authors found small but significant post-intervention and follow-up effects in reducing aggressive behavior in high risk youth who attended programs which much like EiE-L focused on developing youths’ social and relationship skills as well as adaptive responses to provocative situations. More recently, in their meta-analysis of prevention studies aimed at enhancing social-emotional skills, Durlak et al. (2011) identified a small but significant overall effect on problem behavior immediately following treatment, which was maintained at the six-month follow-up. While the primary focus of these meta-analyses was to assess the effects of these programs on externalizing behaviors in adolescence, they also identified positive effects of social-skills based programs on school exclusion, as well as positive outcomes, such as social and communication skills, interpersonal relationships, and school performance. Together these findings suggest that an intervention focusing on building interpersonal communication skills as well as more general social skills may be an efficacious approach to reducing problem behavior and related outcomes, such as school exclusion.

## The Current Study

Members of the research team applied for funding to the European Social Fund’s social experimentation call in 2011, with the explicit aim to rigorously evaluate an intervention aimed at reducing fixed-period school exclusion. A significant motivation for this application was to secure funding to conduct a RCT in this area as at the time very few, if any, interventions aimed at reducing school exclusion in the UK were supported by a rigorous evidence base.

For the current study, the explicit research question was: *can an intervention reduce the incidence and/or frequency of school exclusion, or behaviors associated with exclusion, in a high*-*risk population of students?* This reflects the over-arching aim of the study to bring a rigorous research design to bear on an area of social policy that has largely been neglected by all but a few researchers (exceptions are for example, Gazeley et al. 2015; McCluskey et al. 2015). The specific research questions for the study are reported in the study protocol (Obsuth et al. 2014) and are repeated here. As independent evaluators of EiE-L:

i. We focused on understanding whether the intervention would affect the behavior of participants in terms of officially recorded truancy, temporary and/or permanent exclusions.
ii. We explored the possible effect of the intervention on communication skills of participants in terms of their expressive language, understanding, language processing, and/or social communication skills.
iii. We examined the possible effect of the intervention on self- or teacher-reported disruptive behavior of participants.
iv. We examined the possible effect of the intervention educational attainment of participants in terms of GCSE or other formal tests (e.g., SATs).
v. We focused on assessing whether the intervention would affect self-reported and officially recorded delinquent and/or criminal behavior of participants.
vi. We focused on understanding whether the intervention would affect the likelihood of being Not in Education Employment or Training (NEET) once the children complete compulsory schooling.

From these, (i) addresses the primary outcome of the intervention/evaluation; the remaining questions address secondary outcomes. Specifically, points (ii–iii) relate to anticipated intervention effects arising from the intervention theory of change, which is described above, and the over-arching aim to reduce problem behavior. Points (iv–vi) address additional short-, medium- and long-term outcomes, such as educational achievement and arrests, associated with exclusion as detailed in the above literature review.

The preliminary evaluation yielded generally encouraging findings related to its effectiveness. Moreover, an extant literature suggests that interventions focusing on social skills training yield positive treatment outcomes. Building on these findings, we expected that participation in the Engage in Education-London intervention will yield decreases in students’ exclusions (primary outcome) as well as in behaviors associated with exclusions (secondary outcomes).

## Methods

### Trial Design

The current study utilized a cluster-randomized controlled trial (c-RCT), with randomization at the school level, to evaluate an intervention that was funded and delivered independently of the evaluation team. The trial was registered in the International Standard Randomised Controlled Trial register—Number ISRCTN23244695. This cluster variant trial design was necessary because the intervention approach was a mixture of individual and group sessions, so carried with it a high risk of contagion effects.

### Participants and Data Collection Procedure: Schools and Students

All secondary schools in Inner London with a free school meal (FSM) eligibility rate of greater than or equal to 28 % were contacted via letter pamphlets to participate in the study (n = 108). At the suggestion of the EEF, FSM eligibility rates were used to identify high risk schools. EEF, which funded the implementation of the evaluated intervention, specialises in funding educational research in high-risk schools identified based on high FSM eligibility. Twenty-eight additional schools, which also met the 28 % cut-off for FSM, were contacted from Outer London, resulting in the recruitment of 36 schools. Thus out of 136 contacted schools 36 (26 %) agreed to participate. The students who participated in the intervention were nominated by their school according to the following criteria provided by the study team: the student having had (1) previous exclusions; and/or (2) unauthorized absences and/or (3) having engaged in behaviors that could lead to disciplinary measures. All the students in the study were from years 9 and 10 (aged 13/14 and 14/15, respectively) as these are the years that school exclusion peaks in England (Ellis 2013). The aim was to achieve a sample of around 350–400 students in each arm of the study, with around ten to twelve per year in each school.

Recruitment of schools took place from May to September 2013 (for more information see Obsuth et al. 2014). Students were nominated in June and September of 2013. Baseline students’ data was collected from their teachers in June to September and from students in September to October. Post-intervention data collection was completed one month following the completion of the intervention in each Phase; in March–May, 2014 in Phase I schools and June–July, 2014 in Phase II schools, that is until the end of the 2014 academic year (for further details see published study protocol; Obsuth et al. 2014). Notably, the academic year in the UK starts in the first week of September and ends in the third week of July. As baseline data collection was carried out prior to treatment allocation, both students and teachers were blind to their schools’ allocation during the initial assessment. However, due to the nature of the intervention, it was not possible to conceal treatment allocation and thus the students or teachers were not blind to allocation at the post-intervention data collection.

### Treatment Allocation

**Fig. 1:**
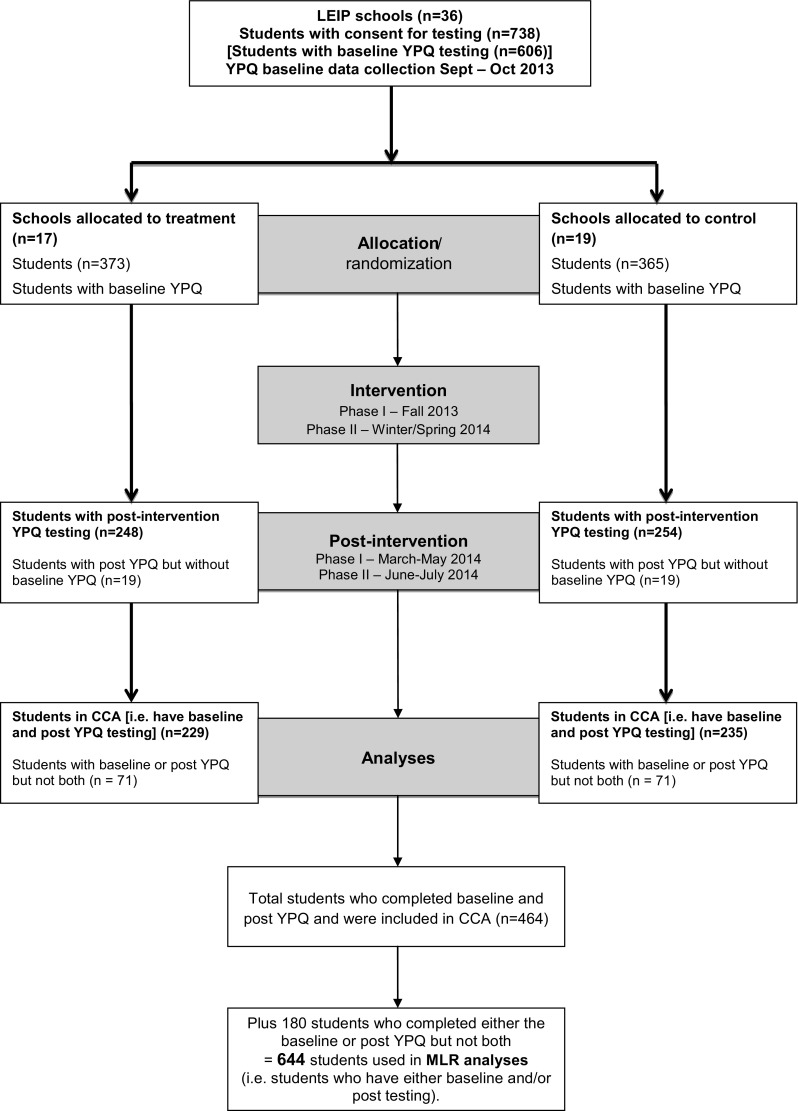
YPQ flowchart

Minimization (Taves 2010) was used to assign schools to the treatment versus control condition. Allocation was undertaken using the MinimPy software developed by Saghaei and Saghaei (2011) based on balancing factors previously identified in other research as being associated with an increased likelihood of exclusion: free school meal eligibility, special educational need status, school size and school composition (mixed vs. single sex), and teacher reported baseline behavior problems. All schools were randomized following completion of baseline data collection. Owing to logistical difficulties with collecting baseline data from all 36 schools in September, allocation was completed in two stages (see Obsuth et al. 2014) by a member of the evaluation team (AS) who was not involved in the data collection and did not access the student data until after randomization had taken place. Overall, 17 schools were allocated to treatment and 19 schools were allocated to control. A total of 382 students had been allocated to the treatment condition in 17 schools and 369 had been allocated to the control condition in 19 schools (total *n* = 751, see Fig. 1).

### Participant Flow

The baseline questionnaire was completed by 300 students (of 373 nominated students) from 17 schools in the treatment condition and by 306 students (of 365 nominated students) from the 19 schools in the control condition (see Fig. 1). Overall, seven students refused to participate in the study, 18 were opted out by their school, five were asked to leave their school via permanent exclusion or a managed move, 23 had left their school and 79 were not available on three or more attempts to complete testing. Teacher questionnaires were available for 539 of these students, including for all 23 who were no longer in school following the summer break.

### Descriptive Statistics

Of the 606 students who completed baseline testing, 430 (71 %) were male (*M* age = 14.05) and 176 (29 %) were female (*M* age = 13.98). More students identified themselves as “Black-African, Black-Caribbean or Black British” (n = 244; 40.3 %) than any other category, with “White British” students comprising the second largest racial grouping (n = 151; 24.9 %). Table 2 presents the baseline demographic characteristics of participants in each group at baseline. With respect to schools, consistent with inclusion criteria, all the schools had a free school meal eligibility rate higher than 28 %, 22 were over 35 % and seven of these had a free school meal eligibility rate of 50 % or more. There were 14 community schools, seven academy converters, six sponsor led academies, four foundation schools, three voluntary aided schools, and two voluntary controlled schools.

**Table 2:** Demographic information for students with baseline YPQ

Allocation	Treatment; n (%) n = 300	Control; n (%) n = 306	Total; n (%) n = 606	*X* ^*2*^ */p* value
*Sex*				
Male	196 (65.3 %)	234 (76.5 %)	430 (71 %)	9.118/.003
Female	104 (34.7 %)	72 (23.5 %)	176 (29 %)	
*Race*				
British European (i.e. White)	90 (30 %)	61 (19.9 %)	151 (24.9 %)	11.572/.116
Other European (i.e. White Non-British)	17 (5.7 %)	14 (4.6 %)	31 (5.1 %)	
Black African, Black Caribbean (i.e. Black)	108 (36 %)	136 (44.4 %)	244 (40.3 %)	
Asian (i.e. Chinese, Vietnamese, Korean etc.)	6 (2.0 %)	8 (2.6 %)	14 (2.3 %)	
South Asian (i.e. Indian, Pakistani, etc.)	31 (10.3 %)	34 (11.1 %)	65 (10.7 %)	
Latin American (i.e. Hispanic)	4 (1.3 %)	5 (1.6 %)	9 (1.5 %)	
Mixed race	29 (9.7 %)	39 (12.7 %)	68 (11.2 %)	
Missing	15 (5 %)	9 (2.9 %)	24 (4 %)	
*Students’ living situation I live with…*				
…my biological mother and father	139 (46.3 %)	124 (40.5 %)	263 (43.4 %)	4.913/.617
…only one biological parent	138 (46 %)	161 (52.6 %)	299 (49.3 %)	
…non-parental care	16 (5.3 %)	16 (5.2 %)	32 (5.3 %)	
Missing	3 (1 %)	3 (1 %)	6 (1 %)	
Other	4 (1.3 %)	2 (0.7 %)	6 (1 %)	
*Were you born the UK?*				
Yes	247 (82.3 %)	246 (80.4 %)	493 (81.4 %)	3.015/.221
No	49 (16.3 %)	5 (1.6 %)	54 (8.9 %)	
Missing	4 (1.3 %)	9 (2.9 %)	13 (2.1 %)	

Given significant baseline differences in the number of girls and boys, participant sex was included in all analyses as a control variable

### Analytic Samples

Separate intent-to-treat analyses were carried out on the data based on the questionnaires completed by the students, their teachers as well as the students’ aptitude in maths and English and official records using different sample sizes for each source of information (see Table 3). Each sample consisted of students who were nominated for the study and for whom data was available either from baseline or post-intervention data collection. Official records were requested for all students who were nominated for the study and for whom relevant information was available.

**Table 3:** Analysis sample sizes by analysis type and data source

Data source	Analysis sample size: students (schools)	
	Complete cases analysis	MLR analysis
Student report (YPQ)	n = 464 (35)	n = 644 (36)
Teacher report (TQ)	n = 424 (34)	n = 685 (36)
CEM aptitude tests	n = 418 (34)	n = 615 (36)
Official records—school exclusion	n = 710 (36)	n/a
Official records—arrests	n = 704 (36)	n/a

### Measures

The primary outcome of the current evaluation was the use of school exclusion as a disciplinary measure. This was assessed via student reports, teacher reports as well as official records. Other, secondary outcomes, that were evaluated reflect previous findings in the literature and as such attempt to measure the negative behaviors associated with exclusion or the mechanisms of change pre-specified by the intervention (see Obsuth et al. 2014). Specifically, communication and broader social skills were identified and measured as the mechanisms of change and key proximal secondary outcomes. Other aspects of interpersonal skills (student–teacher relationships); behavior (antisocial behavior, delinquency, bullying perpetration), and official arrests; as well as in-school disciplinary measures and academic aptitude were also measured and evaluated. These outcomes were evaluated as we expected them to be influenced by the intervention. The outcomes reflect findings that link social skills deficits and communication difficulties to behavioral problems, suggesting that an effect is likely to be found in these areas. Each scale represents a mean score with ranges listed in Table 4.

**Table 4:** Baseline YPQ (n = 606) and TQ (n = 648) mean scores

	Mean (n, SD)	[Range]
YPQ exclusion	1.73 (603, 0.95)	[1.00; 6.00]
TQ exclusion	1.65 (535, 0.72)	[1.00; 4.00]
YPQ other disciplinary measures	2.48 (606, 0.84)	[1.00; 5.58]
YPQ anti-social behavior	1.75 (606, 0.63)	[1.00; 4.40]
YPQ delinquency	1.27 (605, 0.37)	[1.00; 3.45]
YPQ bullying perpetration	1.70 (605, 0.88)	[1.00; 5.67]
YPQ communication	3.46 (604, 0.74)	[1.00; 5.00]
YPQ prosocial thoughts, feelings, behaviors	3.04 (605, 0.85)	[1.00; 5.00]
YPQ student–teacher relationship	3.22 (605, 0.94)	[1.00; 5.00]
TQ other disciplinary measures	2.97 (648, 0.77)	[1.00; 4.83]
TQ anti-social behavior	2.10 (648, 0.60)	[1.00; 4.18]
TQ interpersonal communication	3.63 (612, 0.77)	[1.00; 5.00]
TQ student–teacher relationships	3.37 (648, 0.86)	[1.00; 5.00]
TQ prosocial behavior	2.34 (647, 0.92)	[1.00; 5.75]

*YPQ* Young person questionnaire, *TQ* Teacher questionnaire

The students and teachers completed measures tapping each of the outcomes at baseline and at the post-intervention data collection. At baseline (completed in September/October 2013), students were asked about their behavior in the previous nine months (“… since January”). At the post-intervention assessment, they were asked to recall their behavior in the past four weeks, which corresponded with the month after the intervention had finished. The choice of different recall periods was a pragmatic choice—to extend the recall period further would have meant an overlap with the intervention period. As a result, unless stated otherwise, questions which were rated on a five or six point scale asked respondents to rate the frequency of their behavior, with the lowest score being “never” and highest score being “almost every day” at baseline or “every day” at the post-intervention assessment.

Students completed the “Young person questionnaires” (YPQ), a paper and pencil questionnaire, consisting of 144 questions rated primarily on Likert Scales or yes/no questions tapping into behaviors, emotions, relationships with peers and teachers, as well as communication skills. Notably not all of these questions were utilized as outcome measures as we aimed to collect a wide range of psycho-social behavioral information to gain a better understanding of this unique sample. The duration of the administration of the questionnaire was 30–40 min. In addition the students completed a standardized computerised measure of their academic aptitude (described below). Assessments were completed at school sites, facilitated by a team of 15 temporary research assistants that were recruited and trained to administer the survey and computer testing.

Teachers completed the “Teacher questionnaire” (TQ), which comprised questions tapping similar constructs as the YPQ. It consisted of 39 questions in order to minimize the time of completion to approximately 3–5 min. The intervention provider also provided documents for each group and one-to-one session—referred to as a session plan summary, which summarised the planned content of sessions, provided rating scales to assess behaviors in sessions, time spent on task, and relevant notes. These were utilized to assess engagement with the intervention.

### Primary Outcome

#### School Exclusion

Students and teachers answered questions asking about the frequency of 14 different school disciplinary measures each rated on a six-point scale ranging from “never” to “every day”. Two questions covered the frequency of “fixed-period exclusion” and “suspensions”. We included both terms as they are commonly used in practice, but not always interchangeably. These were used to create a dichotomous outcome of “excluded” or “not excluded”, where *any* exclusion *or* suspension was coded “1” and those reporting ‘never’ to both questions were coded as “not excluded”.

Official records of school exclusions from the National Pupil Database (NPD) of the DfE, UK were also requested. The NPD is a census of all school students in England. Data on exclusions are collected by schools for every student for each term of the school year, with schools asked to specify the type of exclusion and (if applicable) the length of exclusion and the date(s) the exclusion took place. This information is then passed on to the DfE who release aggregated data on exclusions annually. We requested NPD data for 714 students who had consented to allow official records to be requested. Data were returned for 260 students who were reported by their school to have experienced at least one fixed-period exclusion in the 2013/2014 academic year. This period was set to be 6 weeks following the completion of the intervention in each Phase; it constituted the maximum amount of time between the end of the intervention in Phase II schools in May 2014 and the end of the school year.

### Secondary Outcomes: Interpersonal Skills

#### Interpersonal Communication

Students completed a 24-item communication skills measure, with questions such as “can you talk to teachers”, and “can you remember instructions that people tell you?”. Each item was rated on a five-point scale ranging from “1-Never” to “5-All the time”. The tool was developed by I CAN and has been utilized in the pilot evaluation by the DfE (Ellis 2013). It aimed to capture students’ perception of their communication skills in the four areas of communication; understanding, language processing, expressive language, and social communication. The internal consistencies were α = .93 and α = .95 at baseline and at post-intervention, respectively. The teachers completed three of the 24 questions selected by I CAN as tapping the students’ ability to converse effectively. The internal consistencies were α = .73 and α = .84 at baseline and post-intervention assessment respectively.

#### Prosocial Skills

Students completed eight questions tapping overall social skills (behaviors, emotions and thoughts); three items tapped prosocial behaviors adapted from the Social Behavior Questionnaire (SBQ; Tremblay et al. 1991), and five items were adapted from the Interpersonal Reactivity Index (IRI; Davis, 1980): two tapped prosocial emotions (empathy), and three tapped prosocial thinking (perspective taking). Each item was rated on a 5-point scale ranging from “1-Never” to “5-Always” both at baseline and post-intervention assessment. The internal consistencies were .63 and .70. Teachers rated four questions tapping students’ prosocial behaviors. These were originally adapted for teachers from the Social Behavior Questionnaire (SBQ; Tremblay et al. 1991) for the z-proso project (Eisner and Ribeaud 2007). Each item is rated on a 5-point scale. The internal consistencies were α = .83 and α = .80.

#### Student–Teacher Relationship

Students were asked four questions adapted from the What’s Happening In this School Questionnaire (WHSQ; Aldridge & Ala’I, 2013) which tapped their relationship with their teacher, for example, “Teachers support me when I have problems”. The internal consistencies were α = .81 and α = .85. Teachers completed four questions tapping their relationship with the students which were adapted from the z-proso project (Eisner and Ribeaud 2007) and demonstrated good internal consistencies (α = .72 and α = .71).

### Secondary Outcomes: Behaviors

#### Antisocial Behavior

Students completed the adolescent version of the Misbehavior in School (MISQ) measure, developed for this study by the LEIP team to assess teachers’ ratings of students’ behavior at school. It was designed to measure different types of misbehavior, which, according to the DfE (2012), were the most common reasons for exclusion. The measure taps a wide range of behaviors, including persistent disruptive behavior, violence, and inappropriate sexual behavior, rated on a six point scale. The adolescent version consists of 10 items rated on a six-point scale. The internal consistencies at baseline and post-intervention were α = .78 and α = .82. These items mirror those used in the teacher version, however, the language was adapted for students based on feedback from teachers who reviewed the questionnaire during the pilot stage. The teacher version consists of 11 items with internal consistencies of α = .78 and α = .77.

#### Bullying Perpetration

Students were asked three questions tapping the extent to which they engaged in bullying behavior. The items were adapted from a standardized measure of bullying developed by Olweus (1996). The measure assesses three types of bullying: physical, psychological and social exclusion; one question for each type, using a six-point scale. The reliabilities were α = .73 and α = .75.

#### Delinquency

Students completed an 11-item measure tapping their self-reported frequency of substance use (4 items) and delinquency (7 items) using a five-point scale. This measure was adapted from the z-proso project (Eisner and Ribeaud 2007) in which it has demonstrated good reliability. The internal consistencies were α = .80 and α = .92.

#### Arrests

Official records were requested from the Metropolitan Police related to arrest records of all the nominated students in the study for whom we had the required information and parental consent. In the first instance, arrest records were requested for the same students as the NPD data with the exception of ten students for whom the necessary information for data linkage (i.e., date of birth and sex) was not yet available. When this information became available, data was requested but not received. These students were therefore excluded from the analyses.

### Secondary Outcomes: Academic Aptitude

#### Academic Aptitude Measure

Each student completed a computer-administered measure developed by the Centre for Evaluation and Monitoring (CEM) at Durham University. This measure is an adaptive, curriculum free assessment of the students’ aptitude in maths and English (Tymms and Coe, 2003). Year 9 students were administered the MidYIS test and year 10 students were administered the YELLIS test. Based on the general school population who was administered the computer-based test in the 2012/13 school year, the Rasch Person Reliabilities (Model Reliabilities) were 0.892 for the vocabulary subtest and 0.941 for the mathematics subtest.

### Secondary Outcome: Other Disciplinary Measures

Students and teachers completed an instrument tapping the frequency and variety of school disciplinary measures. The 14 items comprising this measure included the most frequently used school disciplinary measures reported by the DfE (2012), including school exclusion, or extra homework. Each item of the scale was rated on a six-point scale. As mentioned above, two of the items were utilized to measure school exclusion. Mean scores were calculated for each informant based on the remaining 12 disciplinary measures and utilized for the analyses to tap “other disciplinary measures”. This scale captures the diversity of disciplinary measures self-reported as being used against the adolescent. The internal consistency of this scale was α = .84 and α = .88 for the student reports and α = .85 and α = .89 for the teacher reports at baseline and post-intervention, respectively.

### Intervention Attendance, Engagement, and Fidelity

In the current study, we assessed three aspects of implementation: intervention attendance, participants’ engagement with the intervention, and treatment fidelity reported by the providers (Durlak and DuPre 2008). Despite minor adaptations in two of the schools due to scheduling problems, the intervention provider reported that the program was delivered in all schools as planned and intended. Of the 320 students in treatment schools and still available in the same school at the beginning of the intervention, 47 students did not attend any group sessions and 40 did not attend any one-to-one sessions; 273 students attended at least one (of 12) group sessions (*M* = 6.85; median = 8); 280 attended at least one of 12 one-to-one sessions (*M* = 6.83; median = 8); and seven students attended all 24 sessions. A total of 208 (65 %) students met the sufficient attendance criteria defined by the intervention provider—they attended five group sessions *and* six one-to-one sessions. The intervention as planned also included home-visits and telephone calls to participants and their family. This resulted in eleven home-visits and 164 telephone calls being made.

Program evaluation research suggests that interventions that are delivered in a manner that promotes engagement in the treatment process yield larger intervention effects. Such built in engagement efforts are particularly important in high-risk and hard to reach populations (e.g., Andrews and Bonta 2006). Mindful of this, we collected information related to the students’ engagement with sessions. To this end, after each session core workers rated the students’ behavior (compliance) in each session on a 5-point scale ranging from 1 (excellent behavior, no disruptions) to 5 (very poor behavior, continuous disruptions). They also rated the amount of time students spent off/on session task and engaged with the content of the sessions, using a 5-point scale, ranging from 1 (80–100 %) to 5 (0–20 %). Conceptually this is a mixture of content covered, behavior and perceived engagement so we treated this as an overall measure of “engagement”. Core workers rated behavior as generally good (*M* = 3.49; *M* = 4.31) and engagement as high (*M* = 3.78; *M* = 4.27 in group and one-to-one sessions, respectively).

### Statistical Analyses

Multilevel models are generally recommended when assessing the effects of programs in cluster randomized controlled trials (Raudenbush 1997). In order to determine whether a multilevel approach should be used we considered the level of intraclass correlations (ICC) for each outcome needed to produce a design effect (DEFF). The ICC is a measure of the proportion of variance in an outcome attributable to differences between groups, in our case schools. The DEFF is the function of the ICC and the average cluster size; DEFF = 1 + (*m* − 1) *ρ*, where *m* is the average cluster size and ρ is the ICC (Campbell et al. 2004). An ICC of .05 is considered large enough to warrant the use of a multilevel approach (Muthén and Satorra 1995). Thus, when ICCs were large enough, the analyses were conducted via intent-to-treat multilevel logistic regression models (primary outcome of school exclusion) and multilevel linear regression models (secondary outcomes). In these models, intercepts were allowed to vary by school to account for between-school variability in outcomes. The student reported outcomes (primary and secondary) and arrests did not have sufficiently large ICCs. Therefore the analyses related to these outcomes were conducted via single level intent-to-treat logistic regression models and single level linear regression models.

All models were estimated in *Mplus 6.11* (Muthén and Muthén 2010), using maximum likelihood estimation with robust standard errors (MLR). MLR provides maximum likelihood parameter estimates to address missing data and utilizes robust standard errors to account for non-normality of outcome variables. It provides unbiased parameter estimates provided that data are missing at random, meaning that the missing values are not related to probability of missingness given the variables in the model (MAR; Little and Rubin 1987). MAR is not empirically testable because it would require the missing values to be known. It is, however, possible to test whether data are missing completely at random (MCAR). This is a stricter assumption and means that missingness is unrelated both to the unobserved missing values and to the observed values of the variables in the model.

To assess whether allocation was associated with missing data/attrition, we carried out logistic regression models where the outcome was whether or not we were able to collect post-intervention data (where 1 = yes). The results showed that students subject to school exclusions at baseline and who engaged in higher levels of moral neutralization, were less likely to be observed post-intervention when compared to those with lower levels of each of these measures. Students who were “white British” or reported high levels of anxiety/depression were also more likely to be missing post-intervention assessments compared to “non-white” students or those with low levels of anxiety/depression. Importantly for our analyses, allocation was not associated with attrition. Results from complete case analyses (CCA; not tabled) were also carried out and did not differ markedly from those reported here. All models were carried out on the intent-to-treat basis and estimated controlling for student sex and baseline values of the evaluated outcome. To keep the number of predictors in the model to a minimum, the randomization variables were not included as covariates.

### Ethics

The project and the consent procedure described below were approved by the Institute of Criminology Ethics Review Committee on the 20th of May 2013. Following identification of the students, ‘opt out’ consent was sought from parents. After approximately 815 letters were sent, 26 parents/guardians opted their child out of the study. Assent was also sought from the students. The study information section of the assent form was read out to them to ensure their full understanding. Thirteen students did not assent to participation, thus their information was not used in any analyses.

## Results

Table 5 outlines the proportions of students who were excluded from school at least once based on self-reported, teacher reported and officially recorded information with reference to the baseline and post-intervention period.

**Table 5:** Proportions of school exclusions at baseline and post-intervention

	Treatment; n (%)	Control; n (%)
*Student report*		
Baseline	168/297 (56.6 %)	168/306 (54.9 %)
Post-intervention	124/249 (49.8 %)	101/254 (39.8 %)
*Teacher report*		
Baseline	177/314 (56.4 %)	205/328 (62.5 %)
Post-intervention	54/217 (24.9 %)	60/232 (25.9 %)
*Official records*		
Baseline	139/363 (38.3 %)	117/351 (33.3 %)
Post-intervention	35/363 (9.6 %)	22/351 (6.3 %)

For student and teacher reports the baseline reporting period was 9 months and post-intervention period was 4 weeks. For official records the baseline period spans one school year (2012/2013) and the post-intervention spans 6 weeks following the intervention

### Intra-class Correlations of Outcomes

The unconditional ICCs for student reported outcomes ranged from 0.002 to 0.028; and 0.024 and 0.015 for the CEM verbal and maths outcomes, respectively. For the teacher reported outcomes these were higher, ranging from 0.062 to 0.211. The ICC for official records of exclusion was 0.050 and for arrests 0.009. The small ICC for student self-reports suggest that there was little between school variation in student-reported outcomes. Similarly, the between school variation in post-intervention arrests was negligible. For this reason, all analyses examining the effects of the intervention on student-reported outcomes as well as arrests were carried out as single level models. All remaining analyses, examining teacher reported outcomes as well as official records of exclusion were carried out as multilevel models.

### Treatment Effects

#### Primary Outcome

The results for the primary outcome of school exclusion based on each source of information are presented in Table 6. Contrary to expectation, students in the treatment condition were significantly *more* likely to self-report being temporarily excluded from school than those in the control schools [*OR* = 1.470, SE = 1.038, *p* = .038; 95 % CI (0.021–0.748)] following the treatment. The estimates based on teacher reported exclusions (OR = 1.022) as well as official records of exclusion (OR = 1.444) paralleled this direction of findings, however, these did not reach statistical significance.

**Table 6:** Logistic regression results for the primary outcome—school exclusion—YPQ, TQ, official records

	Student report	Teacher report	Official records
Analysis sample size: students (schools)	644 (36)	685 (36)	710 (36)
Baseline			
*OR*	2.055	1.535	2.784
SE	0.193	0.263	0.300
*p*	0.001	0.103	0.001
Treatment			
*OR*	1.470	1.022	1.444
SE	0.185	0.364	0.389
*p*	0.038	0.951	0.344
Sex			
*OR*	0.857	1.200	1.466
SE	0.204	0.295	0.350
*p*	0.452	0.536	0.274
Thresholds			
*OR*	2.075	4.495	30.386
SE	0.172	0.310	0.351
*p*	0.001	0.001	0.001

Unconditional between-school variance (ICC) for the primary outcome was 0.028 for YPQ, 0.134 for TQ, and 0.050 for official records of exclusion

#### Secondary Outcomes

Next, we assessed treatment effects on secondary outcomes. There were no statistically significant differences between the students in the treatment versus control condition on any of the adolescent reported outcomes tapping interpersonal (*b* = −0.150 to −0.026), behavioral (*b* = 0.086–0.035), academic (*b* = 0.079 and 0.138) or other disciplinary measures (*b* = 0.160; see Table 7). Similarly, results based on the teacher reported information revealed no statistically significant differences between the two groups on interpersonal (*b* = 0.133 to −0.208), behavioral (*b* = 0.310), or other disciplinary measures (*b* = 0.041; see Table 8).

**Table 7:** Results for secondary outcomes reported by students; n = 464 students, 36 schools

	Comm skills	Pros skills	Teach rel	Anti-soc	Bully perp	Delinq	CEM verbal	CEM maths	Disc
Baseline									
*Est*	0.382	0.518	0.471	0.437	0.351	0.521	0.685	0.641	0.337
SE	0.052	0.050	0.043	0.046	0.056	0.114	0.058	0.058	0.045
*p*	0.001	0.001	0.001	0.001	0.001	0.001	0.001	0.001	0.001
Sex									
*Est*	−0.074	0.062	−0.262	−0.052	−0.105	−0.075	−0.404	0.049	−0.155
SE	0.043	0.092	0.089	0.040	0.073	0.047	1.599	1.449	0.068
*p*	0.086	0.497	0.003	0.190	0.146	0.106	0.800	0.973	0.022
Treatment									
*Est*	−0.075	−0.042	0.024	0.043	0.028	0.032	1.386	2.425	0.124
SE	0.043	0.084	0.077	0.043	0.068	0.051	1.430	1.518	0.065
*p*	0.079	0.620	0.753	0.320	0.683	0.524	0.333	0.110	0.055
Intercept									
*Est*	2.374	1.777	1.659	1.328	0.938	0.697	29.528	27.640	1.139
SE	0.289	0.199	0.158	0.151	0.092	0.143	5.580	5.228	0.110
*p*	0.001	0.001	0.001	0.001	0.001	0.001	0.001	0.001	0.001
*b* (treatment)	−0.150	−0.042	−0.026	0.086	0.035	0.056	0.079	0.138	0.160

*Est* unstandardized estimate, *SE* standard error; Comm skills—interpersonal communication skills; Pros skills—prosocial skills; Teach rel—student–teacher relationship; Anti-soc –antisocial behavior; Bully perp—bullying perpetration; Delinq—delinquency; CEM verbal—verbal aptitude; CEM maths—maths aptitude; Disc—other disciplinary measures. Each column represents a different model, and rows represent variables in those models. Effect sizes are reported as standardized mean differences *(b)* for the single level treatment effect; *b* is standardized with respect to the outcome variance giving the standardized mean difference

**Table 8:** Results for secondary outcomes reported by the teachers; n = 685 students, 36 schools

	Comm skills	Pros beh	Teach rel	Anti-soc	Disc
Baseline					
*Est*	0.316	0.172	0.291	0.220	0.262
SE	0.060	0.049	0.054	0.037	0.048
*p*	0.001	0.001	0.001	0.001	0.001
Sex					
*Est*	0.168	0.164	0.101	0.047	0.034
SE	0.078	0.092	0.087	0.054	0.065
*p*	0.030	0.074	0.247	0.384	0.598
Treatment					
*Est*	−0.072	−0.066	0.027	0.063	0.009
SE	0.138	0.115	0.102	0.079	0.099
*p*	0.600	0.567	0.790	0.421	0.924
Intercept					
*Est*	2.591	1.385	2.378	0.992	1.111
SE	0.247	0.136	0.216	0.086	0.145
*p*	0.001	0.001	0.001	0.001	0.001
*b* (treatment)	−0.208	−0.270	0.133	0.310	0.041

Effect sizes are reported as standardized mean differences *(b)* for the multilevel treatment effect

Notably, all but one of the non-statistically significant estimates related to social and behavioral outcomes indicated negligible *decreases* in positive and *increases* in negative behaviors and skills in the treatment group compared to control group, contrary to what was hypothesised. The one exception was teacher reported communication skills, which suggested non-statistically significant increases in favour of the treatment group following the intervention.

Results from the analysis assessing the differences in arrests (see Table 9) revealed no statistically significant effect of treatment on arrest four-months post-intervention (OR = .731). The rate of arrests following the intervention was comparable in the two groups.

**Table 9:** Logistic regression results for official arrest records—4 months post-treatment arrest; n = 704 students, 36 schools

	Arrest
Baseline	
*OR*	1.402
SE	0.058
*p*	0.001
Sex	
*OR*	0.793
SE	0.260
*p*	0.371
Treatment	
*OR*	0.730
SE	0.230
*p*	0.172
Threshold	
*OR*	7.257
SE	0.176
*p*	0.001

This model is estimated including the 704 students for whom official records of arrests and sex were available. Single level model due to ICC = 0.009

## Discussion

How to address student misbehavior is a problem as old as schooling itself. One current strategy in the UK is the use of exclusion from school. Researchers have been concerned with and have called for attention to school exclusion (Gazeley et al. 2015; McCluskey et al. 2015). Owing to this research a lot is understood today about the multiple risk factors for and negative short, intermediate and long term consequences of school exclusions. Programs and interventions have been used to attempt to address these problems, however, to our knowledge rigorous evaluation of the effectiveness of these approaches has been lacking. Evaluation of programs is essential, particularly in the current climate of austerity and reduced government spending, resources should only be directed to programs which are empirically validated and have demonstrated effectiveness. Furthermore, as programs may not only be ineffective but also have the potential to produce harmful effects (Zane et al. 2016) delivering them without rigorous evaluation is risky and unethical. Our research—the first of its kind in the UK—sought to address some of these knowledge gaps by evaluating an intervention aimed at reducing exclusion and problem behavior through improving social communication and broader social skills of students at high risk for school exclusion.

Our results suggested a small but statistically significant difference for self-reported fixed-period exclusion following the intervention. Specifically, at post-intervention, students in treatment schools were *more likely* to self-report that they had been excluded in the previous month than students in comparison schools. These results suggest a potential negative effect on school exclusion. In line with common practice across the field of psychosocial interventions and the ongoing debate about the need/utility of adjustments in trials (e.g., Schulz and Grimes 2005), we did not account for multiple outcomes in reporting our findings. However, as our analyses yielded only one statistically significant result, we would interpret this with caution given that the family-wise error rate will be increased. Nonetheless, although not statistically significant, the teacher reported exclusion data as well as the official records of exclusion revealed the same direction of effects, more exclusions in the treatment schools post-intervention. With respect to the secondary outcomes, our analyses revealed no significant differences between the students in the treatment schools versus control schools on any of the 15 outcomes, including communication skills, prosocial behaviors, student–teacher relationships, antisocial behavior, delinquency, and official arrest records.

Iatrogenic and null effects are not uncommon in prevention research and researchers have pointed out that understanding of what causes harm in an intervention is as important as understanding what works in order to improve intervention theory and practice (e.g., McCord, 2003). In a recent review of systematic reviews on harmful effects of crime prevention programs, Welsh and Rocque (2014) identified three key reasons for harmful effects: (1) theory failure; (2) implementation failure; (3) and deviancy training. These are also considered useful for understanding null effects, therefore we considered each reason in turn below.

The theory of change identified by Catch22 for the EiE-L program rested on the link between impaired communication/social skills and behavior, which may lead to exclusions. Although previous studies have suggested links between social-emotional deficits and behavior problems (e.g., Durlak et al. 2011), our findings suggest that targeting social communication and broader social skills may not be an effective strategy to intervene with students at the *highest* risk for school exclusion. Even with a short post-intervention follow-up period where we would expect to find the strongest positive relationships, positive effects on communication and prosocial skills were not observed. Further, social communication and broader social skill deficits may simply be symptomatic of other issues rather than causally related to behavior. It is possible, for example, that executive functioning deficits may be confounding this link (see e.g., Hughes et al. 2009). If so, efforts to improve broader social skills would be ineffective if these cognitive deficits were not being addressed at the same time.

The importance of implementation quality and its impact on the success or failure of interventions has been widely demonstrated (e.g., Durlak and DuPre 2008; Wilson et al. 2003). However, measuring implementation quality is difficult because it is a multifaceted construct, which includes the quality of program delivery as well as participant involvement (Bishop et al. 2014). Measures of program delivery include: evaluation of adherence to a curriculum; training of staff; and time spent on/off task in sessions. Participant involvement can include: consideration of attendance or dosage, participants’ engagement, and behavior in sessions. Therefore, with respect to implementation our data shows two areas for concern. First, there is evidence of low exposure to treatment for those in treatment schools. Specifically, from an intended twelve individual and twelve group sessions, the average number of sessions attended was 6.85 for one-to-one sessions and 6.83 for group sessions. This suggests that there were fewer opportunities for the intervention to actually take place than was intended. Given the high-risk sample, low attendance is an understandable challenge, but one that intervention providers should anticipate and for which they should prepare. Problems with attendance and engagement are perhaps more likely when dealing with a high-risk sample.

Second, although Catch22 believe that content was delivered as intended, in other words with high fidelity, and no significant variations were reported by the intervention team, a review of weekly EiE-L session progress and action logs revealed that core workers encountered a variety of organisational and logistical difficulties in several schools. Furthermore, the intervention design allowed for home visits and telephone calls to the students’ families, which could have been employed to address attendance and engagement problems. However, comparatively few phone calls were made (n = 164) and only eleven home visits were completed. For illustration purposes, 47 students did not attend any group sessions at all. If one phone call had been made for each session that these 47 students alone did not attend, then a total of 564 phone calls would have been made. This seems like a missed opportunity for re-engaging youths and their families in the program and in their education more generally. The intervention provider appears to have had low expectations for the attendance and engagement of students, despite aiming to alter their behavior. Poor attendance (dosage) as well as engagement and other relevant factors may affect the impact of interventions (Rothwell 2005).

The third often cited reason for harmful or null treatment effects, deviancy training, has been observed in interventions targeting students with severe behavior problems (e.g., Dishion and Tipsord 2011). The process is often referred to as “deviant peer contagion” (Dodge et al. 2006), “delinquent spiral” (Cécile and Born 2009), or “drift into deviance” (Dishion et al. 1999). Several mechanisms underlying the negative effects of treating students and their behavior problems in a group format have been described. The predominant view is that students in these situations encourage each other’s behaviors through mutual participation and deviant or antisocial talk or verbal statements which are seen as potent sources of reinforcement (Dishion and Tipsord 2011). Developmental psychologists have suggested that children and students who have experienced social exclusion and rejection are more likely to be susceptible to negative group influences in search of belonging; conforming to what the group does and how the majority or a strong individual behaves; and in order to achieve social status (e.g., Gifford-Smith et al. 2005). This is particularly true during adolescence, when students are generally more susceptible to peer influences (e.g., Menting et al. 2015). It is therefore possible that the negative group influences cancelled out the possible positive intervention effect and hence yielded null post-intervention findings.

According to Moon et al. (2010), “null results, or no differences between groups, are an important but often hidden aspect of scientific inquiry, potentially contributing as much to knowledge as superficially more ‘successful’ studies that support hypotheses and provide positive advances to understanding” (p. 482). There are two possible methodological factors that may account for no effects and so must be considered: measurement problems and statistical power. In terms of measurement, sub-scales from well-validated measures were used and these scales had high reliability in the study sample. In terms of statistical power, the study operated under practical constraints that limited the number of schools/participants. The study was planned on the basis of being able to detect standardized differences of around d = .35 (see Obsuth et al. 2014). The models achieved statistical power very close to that planned and even on occasion bettering it (owing to smaller ICCs than anticipated). Moreover, with the exception of one (student–teacher relationships, from adolescent report data) of the total of 18 tested models, all of the estimates were pointing in the direction of iatrogenic rather than positive intervention effects. This leaves two additional possible reasons for no effects. First, that the intervention was not implemented well enough to result in any change on these outcomes, or second, that the intervention was implemented well, but did not affect the students’ behavior in a meaningful enough way. The relatively high scores on our two measures of implementation quality, students’ behavior in sessions and time spent on-task, suggest that an adequate implementation quality was achieved. However, in the context of relatively low attendance, another often utilized measure of implementation quality (e.g., Durlak and DuPre 2008), it is possible that the treatment providers did not achieve a desired engagement with the program which may have allowed participants to benefit from it. These possibilities are further explored in sub-group analyses presented in Obsuth et al. (in press).

This study suggests that short-term school-based interventions that have not been well-integrated into school provision, or are otherwise ‘external’ to the school, are unlikely to be successful in changing students’ behavior, particularly students who have already had difficulties at school. Whils not ‘news’ to researchers in this field, the intervention approach set out here is one frequently encountered in the real world, particularly when working with students who are marginalised (e.g., Cooper et al. 2007). Implementation of behavioral interventions with high-risk adolescents needs to be carefully managed and teachers need to be on-board from very early on (Nation et al. 2003; Theimann 2016). Adolescence is a developmental period characterised by marked and rapid biological, cognitive, emotional and social changes. As a result, it has been identified as the second major ‘window’ of opportunity for positive changes as well as sensitive period for risk, next in significance to early childhood development (e.g., Moretti and Peled 2004). Given the structural and functional changes in their brain’s dopaminergic system responsible for the regulation of socio-emotional processes, students are more likely to engage in risk-taking behaviors, or behaviors with potential for harm to self and others, such as delinquency, substance use, dangerous driving, than younger children or adults (e.g., Steinberg 2015). They are generally more susceptible to peer influences and are more likely to engage in risk-taking behaviors and/or delinquency in the presence of peers (e.g., Menting et al. 2015). Interpersonally, students expand their social circles; spend more time with peers and form their first serious romantic relationships. In their apparent striving to establish a new balance between dependence on their carers for support and their autonomy or independence (e.g., Oudekerk et al. 2015), it may appear that they no longer rely on their parents and other significant adults (such as teachers, mentors) for help and support. However, evidence suggests otherwise. Recent studies highlight the importance of positive student–teacher relationships and strong school bonds in healthy adolescent development (Silva et al. 2016; Theimann 2016). For example, Theimann (2016) found that positive student–teacher relationships in the context of positive bonds to school were related to lower rates of delinquency in students from age 13 to 16. A meta-analysis by Wilson et al. (2003) found that interventions delivered by teachers were more effective than those delivered by offsite providers. Anecdotal evidence from the EiE-L core workers indicated that in some instances schools informed students that they were enrolled on the intervention because they were the “worst kids”; this may not only hinder any engagement in intervention but also jeopardise the teachers’ relationships with the students and thus contributed to negative effects. Adolescence is a volatile transitional period and more care should be taken to consider this when introducing and delivering any intervention. Moreover, positive experiences and relationships within schools (both with peers and teachers) have been well documented (e.g., Layard et al. 2014; Silva et al. 2016; Theimann 2016), therefore the tendencies to exclude are particularly troubling.

Rates of exclusion were alarmingly high for the students in this study, with 30–50 % (based on official records and questionnaires, respectively) receiving a temporary exclusion in both treatment and control schools in the year prior to the study. Moreover, nine per cent of students in treatment schools and 6 % of students in control schools experienced an officially recorded exclusion in the six week period immediately following the intervention. These rates were much higher based on teacher and adolescent reported exclusions. This discrepancy may reflect the often described problem of unrecorded/unreported school exclusions (e.g., Gazeley et al. 2015). Furthermore, multiple exclusions were not uncommon within the students who were included in our analyses, suggesting that the study had indeed correctly sampled those at the greatest risk of exclusion.

The rates at which exclusions occurred among our sample suggest that schools are struggling to deal with a significant proportion of students for whom they are responsible. The need to think differently about how to manage students with problem behavior is clear. An approach that emulates the collaborative emphasis of the Communities that Care (Kim et al. 2015) or Positive Behavioral Interventions and Supports (e.g., Horner and Sugai 2015) models, with several care providers sharing the responsibility for tackling a problem, may prove fruitful in this respect. These approaches suggest that, to achieve effective and lasting change in students’ interactions, behaviors, and emotions, the whole school needs to be addressed, with the empirically supported view that those with greatest need will benefit most (Tolan et al. 2004). It should be noted that such approaches incorporate greater assistance and resources for those with the greatest needs, but within an inclusive, whole-school framework. Programs employing similar principles of care have been evaluated and revealed positive effects on youth behavior, delinquency as well as school exclusions in the UK and elsewhere (e.g., Pritchard and Williams 2001). Moreover, in further support of the importance of a school-wide supportive context, recent longitudinal research (Layard et al. 2014) revealed that good experience of education was more important than what individuals learned at school for good outcomes in life 40 years later. This study highlights a need for a shift from a focus on achievement alone to a focus on healthy child development in schools. Efforts should be made to identify feasible alternatives building on principles of inclusion and healthy emotional development. Policy change may be required to allow schools sufficient autonomy to deliver such models.

As with any project, this study has some limitations which were mainly related to scheduling. Information was collected from participants at particularly busy times at the beginning and end of the school year. This contributed to initial difficulties completing all of the baseline data collection in September and led to the two phase design. As we noted above, the providers self-reported program fidelity, in other words that the sessions were delivered as intended. Provider reports of problems in the implementation of an intervention are a common practice in assessing treatment fidelity in behavioral program evaluation research (see for example a recent overview of meta-analyses by Sandler et al. 2014). We have built on the recommendation of Sandler et al. (2014) by broadening the assessment of implementation quality, adding measures of dosage and treatment engagement. However, in this study the relatively low attendance and utilisation of family contact, suggests a gap between provider perceptions of implementation and achieved program delivery. Independent observations of the implementation quality would perhaps lend a better, less biased, insight into the implementation process. However, due to the short time span of the current evaluation (2 years in total) this was not possible in this study. Similarly, our resources and time restrictions did not allow for the collection of information related to therapeutic alliance, which could further help us explain our findings.

Yet, this study also has several important strengths. It is one of the first school-based large-scale independent c-RCTs of a behavioral intervention aimed at high-risk students in the UK. The research involved an innovative approach to fieldwork in recruiting a large temporary fieldwork team in order to collect data more efficiently than would be possible with a smaller but full-time group of fieldworkers. Given the high-risk group, those typically absent from school surveys, a relatively high retention rate was achieved (77 % of n = 606 at baseline), which is notable given the nature of this population: it is well-known that individuals with the highest levels of problematic behavior are at particular risk of non-participation and dropping out (e.g., Eisner and Ribeaud 2007). Questionnaires were administered to multiple informants and official records were secured (for the primary outcome), which allowed the interpretation of findings with greater confidence. An effective collaboration between the agencies involved in the study; namely the Greater London Authority, the Education Endowment Foundation, Catch22, I CAN and the 36 schools; and the research team was developed. We achieved good balance on the key variables identified in the protocol. We also included additional measures that allow us to understand this high-risk group of students and the project allows the possibility of tracking this group over the long-term using administrative data. Finally, we achieved sufficient power to detect small to medium effects.

While this study focused on schools in England, we believe the findings are generalizable to other jurisdictions. The schools included in this study represented deprived areas of London where the social composition does not necessarily reflect that of the rest of the UK or other countries. However, trends in exclusion appear to be relatively universal across countries, with boys, ethnic minority students and those with stated, as well as undiagnosed, learning difficulties being disproportionately suspended and excluded from schools (Achilles et al. 2007; Day-Vines and Terriquez 2008; Losen et al. 2015). Studies focusing on whole-school approaches to tackle school exclusion have had equal success in the US (Bradshaw et al. 2010) and other countries, such as Norway (Sørlie and Ogden 2015). There are significant differences in approach to the provision of public and social services in these two countries, as well as in the historical and cultural context of the integration of ethnic minorities and social classes. However, despite these differences, similar approaches to tackling shared problems appear efficacious. This suggests that research in this area will be applicable to different cultural contexts.

## Conclusion

School exclusion is a significant societal concern which seems likely to have harmful short- and long-term effects on students. Programs addressing this concern have been largely unevaluated. In this study, we employed a rigorous c-RCT design to evaluate an existing intervention which attempted to reduce school exclusion. Providers external to the school delivered communication and social skills training in twelve group and twelve one-to-one sessions. Our study suggested primarily null effects with one negative finding, suggesting that short-term interventions similar to EiE-L, delivered in schools by external providers, may not be an effective strategy for reducing school exclusion and related behavioral problems. The intervention did not foster improvements in school bond, school climate, student–teacher relationships and positive peer influences, all shown to have important effects on adolescent outcomes. Adolescence is a period of rapid developmental changes in many areas, resulting in greater sensitivity to environmental influences (Moretti and Peled 2004). The trend toward school exclusion is paradoxical, given the growing evidence that schools play an important role in students’ academic, cognitive and socio-emotional development both during childhood and adolescence (e.g., Layard, et al. 2014; Silva et al. 2016; Theimann 2016). Therefore, keeping students in education, by providing them with an inclusive school environment, which would facilitate school bonds in the context of supportive student–teacher relationships, should be seen as a key goal for educators and policy makers in this area. The development and evaluation of interventions focusing on improving school environments and fostering a healthy school climate should be a central part of achieving these goals. In this climate, teachers and school management would ideally not view exclusion as a viable behavior management strategy. Examples of such approaches are available and evidence from other countries suggest that they are effective in reducing school exclusion and yield positive socio-behavioral, emotional and educational outcomes (e.g., Bradshaw et al. 2010).
